# Anisotropy of Percolation Threshold of BaTiO_3_-Ni_0.5_Zn_0.5_Fe_2_O_4_ Composite Films

**DOI:** 10.1038/s41598-019-44328-7

**Published:** 2019-05-27

**Authors:** Yu Tang, Ruixin Wang, Yi Zhang, Shun Li, Piyi Du

**Affiliations:** 1 Department of Materials Science and Engineering, College of Aerospace Science and Engineering, National University of Defense Technology, Changsha, 410073 China; 2 Department of Physics, College of Liberal Arts and Sciences, National University of Defense Technology, Changsha, 410073 China; 3 State Key Laboratory of Silicon Materials, School of Materials Science and Engineering, Zhejiang University, Hangzhou, 310027 China

**Keywords:** Magnetic properties and materials, Magnetic properties and materials

## Abstract

A systematic study on the magnetic and electrical percolation phenomena of BaTiO_3_ (BTO)-NiZnF_2_O_4_ (NZFO) composite films is presented in this work with the purpose of simplifying the preparation process of high-performance 1–3-type multiferroic composite films. Results show that the percolation threshold of the composite films depends on the macroscopic dimension of the material. The low-dimensional nature of the composite films results in different percolation thresholds with topological transition in vertical and horizontal directions. BTO-NZFO composite films with a grain size of 15 nm and a thickness of 100 nm exhibited a percolation threshold of 0.18 in the normal direction and a percolation threshold of 0.48 in the horizontal direction. In light of this intriguing feature, a novel multiferroic composite film with 1–3 structure and strong magnetoelectric coupling was easily prepared by a 0–3 process via controlling the NZFO content in the region between two percolation thresholds.

## Introduction

Multiferroic materials have become a research focus because of their special properties, such as magnetoelectric coupling, and manifested great potential in the application fields of high-density storage and fast multi-state response^1–4^. Several strong magnetoelectric coupling systems have been reported since 2004^5–7^, and the magnetoelectric coupling mechanisms including exchange bias and interface control of multiferroic materials also have been revealed, thus broadening our knowledge of the intrinsic properties of such systems^8–10^. Among these mechanisms, the most widely recognized one is the strain coupling mechanism proposed by Eerenstein and Thiele^11,12^, in which strain transmission between lattices can control the magnetic/electrical properties of the material under external electric/magnetic fields. According to this mechanism and the origin of dipole/magnetization response, magnetoelectric coupling effect can be seen as the result of magnetic/electric dipole deflection, which is caused by the lattice strain transmission between the ferroelectric/ferromagnetic phases and the anisotropic deformation in the lattice under an external field. Therefore, 1–3 type multiferroic composites composed of 1D ferromagnetic/electric nanowires embedded in a 3D ferroelectric/magnetic matrix exhibit a stronger magnetoelectric coupling than those with other composite structures^2,5,7–9^. However, compared with 1–3 composites, 0–3 composites, in which 0D particles are dispersed in a 3D matrix, possess obvious advantages of easy controllability and satisfactory repeatability of preparation^13–19^. In this context, it is of great interest and significance to find a novel strategy that can overcome the trade-off between the process and the performance of multiferroic composites and prepare 1–3 multiferroic composites via a 0–3 process.

**Figure 1:**
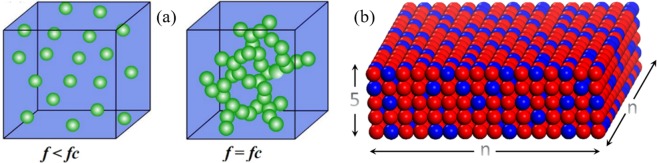
Sketch map of the distribution of two kinds of grains in a composite.

Percolation theory offers the possibility to implement this idea. According to percolation theory^20^, when the content *f* of a constituent phase in the composite reaches a critical value (i.e. the percolation threshold *f*_c_), the particles begin to connect with each other to form a percolation channel as shown in Fig. 1a. Calculated by the equal-radius ball random model (Fig. 1b), the percolation threshold is supposed to be 1 for a 1D system, 0.45 for a 2D system, and 0.16 for a 3D system^21,22^. For a very thin (nanoscale) composite film, the grain connection in the horizontal (in-plane) direction of the film will follow the characteristics of a two-dimensional system, while the connection in the vertical direction (out-of-plane) of the film will follow the characteristics of a 3D system. Therefore, in principle, 1–3 multiferroic composite films in which the nanoparticles are connected out-of-plane and isolated in-plane can be probably formed by a simple 0–3 process, as long as the composition of the ferroelectric-ferromagnetic composite films lies in the region between the two percolation thresholds of 2D and 3D systems.

Nevertheless, this is only a theoretical assumption that needs to be verified experimentally. Subject to limited observation area of transmission electron microscopes and thickness of samples, it is difficult to directly observe the percolation channel that normally connects the entire film. Therefore, a method to demonstrate the anisotropy of multiferroic film percolation thresholds in different directions is also of vital importance and practical significance.

Herein, a classical multiferroic film system, BaTiO_3_ (BTO)-Ni_0.5_Zn_0.5_F_2_O_4_ (NZFO), is utilized to verify the proposed strategy. The films were prepared by magnetron sputtering, which is a conventional process for 0–3 composites. The percolation behavior of the composite films was investigated by analyzing the electrical and magnetic properties under external fields with different directions. The anisotropy of the percolation threshold of the multiferroic composite thin films was systematically characterized for the first time. A novel way of preparing multiferroic composites with excellent performance by a simple process was verified.

## Results

**Figure 2:**
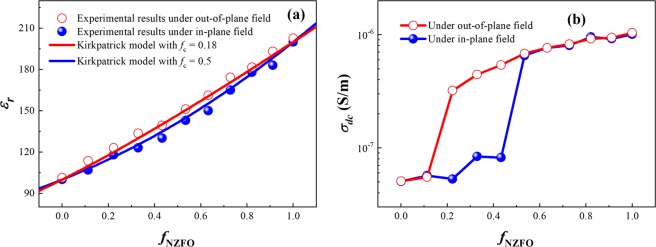
(**a**) Permittivity and (**b**) dc conductivity of the BTO-NZFO composite thin films under out-of-plane and in-plane electric fields as a function of *f*_NZFO_.

Figure 2(a,b) show the permittivity and dc conductivity of the BTO-NZFO composite films under out-of-plane and in-plane electric fields as a function of the volume fraction of NZFO *f*_NZFO_, respectively. As shown in Fig. 2(a), the permittivity of the composite film increases nonlinearly from 100 to 200 as the value of *f*_NZFO_ increases, regardless of the direction of the electric field. However, for the composite films with the ferrite fraction lying between 0.2 and 0.8, the permittivity under an out-of-plane electric field is slightly higher than that under an in-plane field. It is known that the permittivity of the BTO-NZFO composite films conforms with the Kirkpatrick model^23^. In Fig. 2(a), it can be seen that the experimental values of the permittivity of the composite films under the out-of-plane electric field can be well fitted by the theoretical curve when the percolation threshold *f*_c_ is set as 0.18, and the theoretical curve is consistent with the experimental results under the in-plane field when *f*_c_ is set as 0.5. In addition, as shown in Fig. 2(b), the conductivities of the composite films under the both electric fields exhibit a leap at a certain point with increasing *f*_NZFO_. Below and above this point, the conductivity values remain almost the same in both cases. It is noteworthy that the abrupt-change point of the conductivity of the composite film under the out-of-plane electric field is *f*_NZFO_ = 0.16, while that under the in-plane field appears *f*_NZFO_ = 0.42.

The variation in the conductivity of the BTO-NZFO composite films versus NZFO content shown in Fig. 2(b) is a typical evidence of conductivity percolation that usually occurs in the composites composed of the constituents with different conductivities. The abrupt-change point of the conductivity represents the value of *f*_c_, which is the ferrite volume fraction at the time of the formation of conductive paths in the direction of the electric field. It is well known that under an electric field, the free charges inside the material will move to generate current. The direction of the charge migration is from one electrode to the other following the minimum voltage drop principle; that is, the migration paths are always along the paths with smallest total resistance under a constant applied voltage. The results in Fig. 2(b) show that when *f*_NZFO_ = 0.16, a path consisted of low resistivity phases forms out-of-plane, while it forms in-plane when *f*_NZFO_ = 0.42 comparatively. In BTO-NZFO composites, the conductivity of the NZFO phase is much larger than that of the BTO phase. Therefore, the formation of the low-resistance paths means that the NZFO grains between the positive and negative electrodes are able to connect to form percolation channels. When the NZFO volume fraction lies between 0.2 and 0.4, the NZFO grains fully connect out-of-plane, despite they are not yet fully connected in-plane at this time so that the charges cannot find a path with less voltage drop for long-range migration.

**Figure 3:**
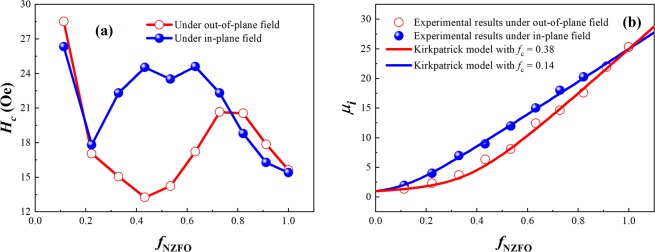
(**a**) Coercivity and (**b**) initial permeability of the BTO-NZFO composite thin films under out-of-plane and in-plane electric fields as a function of *f*_NZFO_.

Figure 3(a,b) show the coercivity *H*_*c*_ and the initial permeability *μ*_*i*_ of the BTO-NZFO composite films under out-of-plane and in-plane magnetic fields as a function of *f*_NZFO_, respectively. As shown in Fig. 3(a), when the ferrite content in the composite films increases, the coercivity of the composite films in the vertical magnetic field decreases from 28.5 Oe to 13.2 Oe in the range of *f*_NZFO_ = 0.113–0.433, thereafter it begins to increase and reaches a peak of 20.6 Oe at *f*_NZFO_ = 0.728, and finally drops to 15.6 Oe as *f*_NZFO_ reaches 1.0. On the contrary, the value of *H*_*c*_ under an in-plane magnetic field increases with increasing *f*_NZFO_ and reaches a peak of 24.6 Oe above *f*_NZFO_ = 0.632, although a decrement from 26.3 Oe to 17.8 Oe can be observed in the range of *f*_NZFO_ = 0.113–0.223. It then begins to decline with the increase in *f*_NZFO_ until *H*_*c*_ = 15.4 Oe at *f*_NZFO_ = 1.0. As shown in Fig. 3(b), the values of *μ*_*i*_ under vertical and parallel magnetic fields both increase nonlinearly from 2.9 to 25.4 with the increase in NZFO ferrite volume fraction. However, when the ferrite volume fraction lies between 0.2 and 0.8, the initial permeability *μ*_*i*_ of the composite films under an in-plane magnetic field is higher than that under an out-of-plane field.

**Figure 4:**
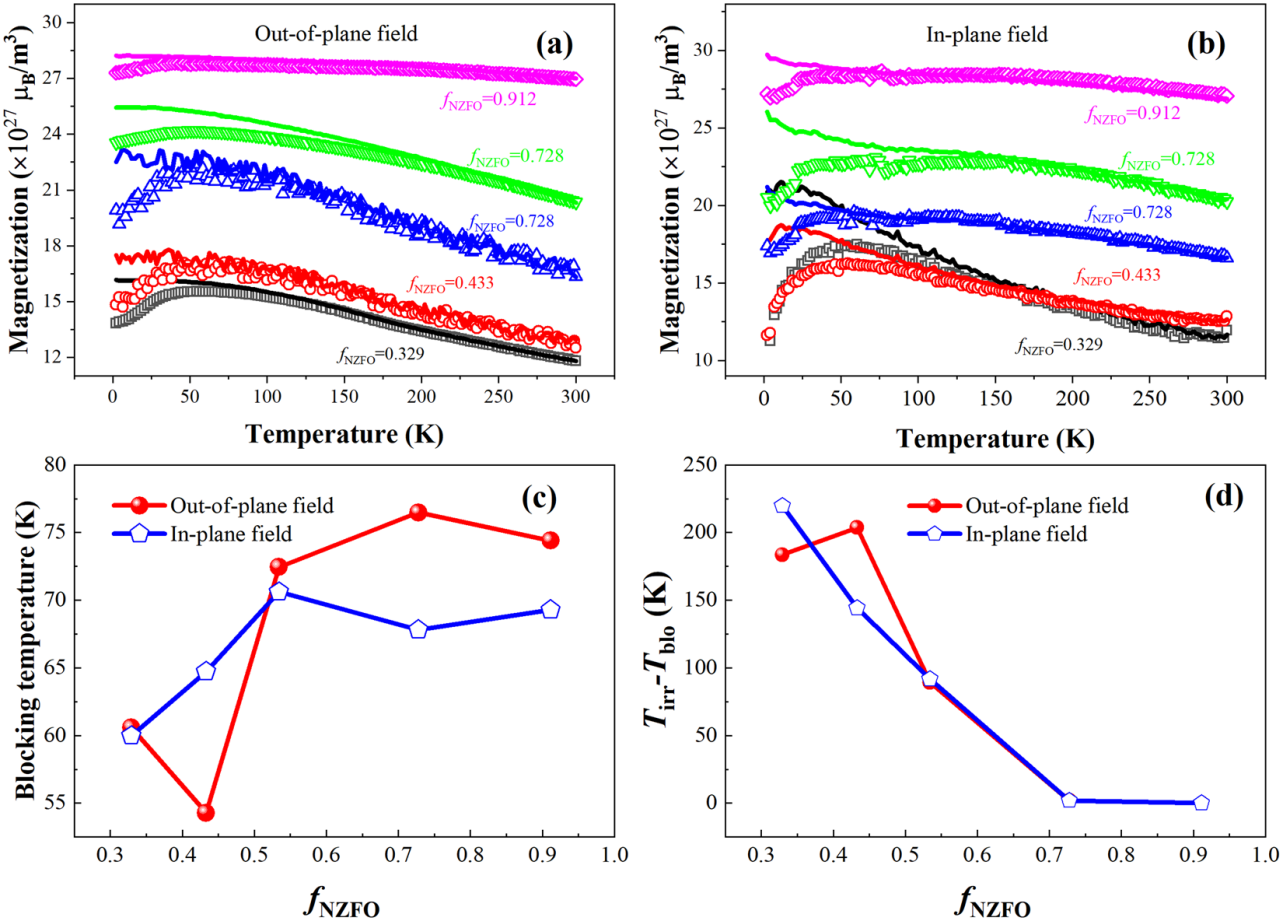
ZFC-FC curves of the BTO-NZFO composite thin films with different ferrite contents under (**a**) out-of-plane and (**b**) in-plane magnetic fields. (**c**) The blocking temperature and (**d**) the value of *T*_irr_ − *T*_blo_ of the composite thin films as a function of ferrite content.

Figure 4(a,b) show the field-cooled and zero-field-cooled (FC-ZFC) curves of the BTO-NZFO composite films with *f*_NZFO_ = 0.329, 0.433, 0.534, 0.728 and 0.912 under vertical and parallel magnetic fields, respectively. The plots of the variation of the average blocking temperature, *T*_blo_, and its difference with the irreversible temperature *T*_irr_, i.e. *T*_irr_ − *T*_blo_, versus *f*_NZFO_ under different magnetic fields are shown in Fig. 4(c,d), respectively. As shown in Fig. 4(c), under an out-of-plane magnetic field, *T*_blo_ of the composite films first increases from 58.4 K to 72.5 K as *f*_NZFO_ increases from 0.3 to 0.5, and then begins to decrease. By contrast, the value of *T*_blo_ first decreases from 60.6 K to 54.3 K with *f*_NZFO_ increasing from 0.3 to 0.4, and then increases until a maximum value of 76.5 K is reached at *f*_NZFO_ = 0.728 under an in-plane magnetic field. As shown in Fig. 4(d), as the ferrite content increases, the value of *T*_irr_ − *T*_blo_ under the vertical external magnetic field remains relatively stable first and then decreases from *f*_NZFO_ = 0.433 to *f*_NZFO_ = 0.728, and finally remains unchanged. The value of *T*_irr_ − *T*_blo_ under a parallel external magnetic field decreases linearly until *f*_NZFO_ = 0.728, and then remains almost unchanged.

It has been documented that in BTO-NZFO composite films, the exchange between the ferrite nanocrystals and the magnitude of induced magnetic flux can change due to the topological changes of the composites, resulting in a percolation transition of the initial permeability and coercivity in such multiferroic systems^23^. When the ferrite content in the composite films is lower than the percolation threshold, the magnetic NZFO grains are dispersed in the non-magnetic BTO matrix. The transmission of the magnetic induction current between the magnetic particles is blocked by the nonmagnetic BTO particles, leading to a small value of the initial permeability of the composite due to the suppression of the equivalent demagnetizing field. Moreover, as there is no interaction occurring between the grains, the coercivity and the anisotropy field of the composite are the average of all magnetic grains. The variation in coercivity and anisotropy field with changing composition of the composite is actually controlled by grain size. Owing to the diffusion during the crystallization process, the grain size of NZFO in the composite films generally increases with increasing ferrite content. Increased grain size renders the average anisotropic energy (represented by *T*_blo_) and the coercivity of the material to decrease with increasing *f*_NZFO_, while the difference between the highest anisotropic energy and the average anisotropic energy (represented by the value of *T*_irr_ − *T*_blo_) does not change.

When the ferrite content in the composite films reaches the percolation threshold, the magnetic NZFO grains are connected with each other, forming a magnetic flux channel and initiating the exchange effect. The formation of the magnetic flux channel causes the initial permeability of the composite to be less suppressed by the equivalent demagnetizing field, and such effect accounts for the nonlinear increase in initial permeability with increasing ferrite content. The exchange effect in a material also tends to impel the coercivity to exhibit the same value with that of the harder interconnected grains. Before NZFO is completely interconnected and a new matrix phase comes into being, as the ferrite content increases, the number of interconnected NZFO grains increases, and the magnetization of hard magnetic grains can be transferred to other grains. The coercivity of the average anisotropic energy of the composite is consequently increased, and the difference between the highest anisotropy energy and the average anisotropy energy is reduced. When the NZFO grains in the composite films are completely interconnected to form a matrix phase, the exchange between the grains has reached saturation, and *T*_irr_ − *T*_blo_ no longer increases with increasing ferrite content. The coercivity and *T*_blo_ of the composite films is once again controlled separately by ferrite grain size, which decreases with increasing ferrite content.

In ligh of linear physical transport, the percolation threshold of the magnetic permeability in a composite can be given by fitting the experimental results to the Kirkpatrick model^24^. Under the out-of-plane and in-plane magnetic fields, the values of *f*_c_ are 0.38 and 0.14, respectively, as shown in Fig. 2(b). The value of percolation threshold can also be directly obtained from the coercivity as depicted in Fig. 2(a); that is, *f*_c_ ≈ 0.4 under vertical magnetic field and *f*_c_ ≈ 0.2 under parallel magnetic field. These results are in good accordance with the phenomena demonstrated in Fig. 4(c,d) that the magnetic induction of the out-of-plane field starts to transfer between the magnetic particles when *f*_NZFO_ ≈ 0.4, and that of the in-plane field can transfer when *f*_NZFO_ < 0.3.

More interestingly, the values of *f*_c_ for the electric and magnetic responses of the composite are different. Precisely speaking, their behaviors are opposite under external fields with out-of-plane and in-plane directions. In fact, such difference is caused by the same transition in the topological microstructure of the composite films as well as the nature of magnetic response. It can be speculated that both exchange and magnetic current are generated in a direction perpendicular to the external magnetic field. Hence, the percolation transition of the magnetic properties of the multiferroic composite under out-of-plane magnetic field is caused by a topological transition along in-plane direction, while the transition under a radial magnetic field is caused by a topological transformation in the normal direction of the films. In addition, since the magnetic induction can cross the non-magnetic barrier layer of thickness ~15 nm^19^, the magnetic percolation threshold in the composite films appears in a much lower ferrite content region than the threshold of electrical performance and geometrical percolation.

## Discussion

**Figure 5:**
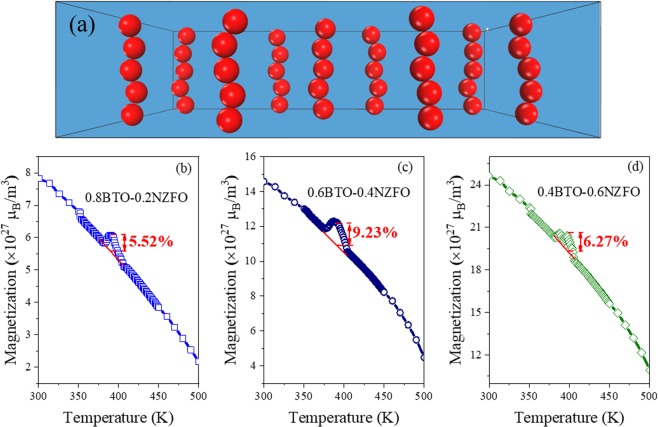
(**a**) The 1–3 structure and the magnetization of (**b**) 0.8BTO-0.2NZFO, (**c**) 0.6BTO-0.4NZFO and (**d**) 0.4BTO-0.6NZFO composite thin films at different temperatures.

The electrical and magnetic properties of the composites (Figs 2–4) confirm the mathematical model simulation of the lattice network (Fig. 1), indicating that the BTO-NZFO composite films possess different percolation thresholds in the normal and radial directions, i.e. 0.16–0.18 and 0.42–0.5, respectively. In low-dimensional BTO-NZFO composite films, the out-of-plane percolation threshold will be lower than the in-plane percolation threshold. This fact implies that the nanowire-like channels connecting the NZFO nanocrystals come into existence in the normal direction instead of the radial direction in the composite. If the composition of the composite films is tailored to lie between the percolation thresholds in two directions (x = 0.2–0.4), some NZFO paths that are arranged in the normal direction and not in contact with each other in the radial direction can be possibly formed in the composite films. Each NZFO path can be approximated as a one-dimensional nanowire in the ferromagnetic phase. Conclusively, it is entirely possible to form a 1–3 structure in which the ferromagnetic phase is 1D nanowire and the ferroelectric phase is a matrix. In other words, the 0.6BTO-0.4NZFO composite thin film prepared by a simple 0–3 process may have a large area of anisotropic contact between the ferroelectric and the ferromagnetic phases as shown in Fig. 5(a), thus giving rise to a magnetoelectric coupling as strong as 1–3 multiferroic composites.

As can be seen from Fig. 5(c), at the Curie point (~375 °C) of BTO where the first-order phase transition from tetragonal to cubic structure occurs, the magnetization of the 0.6BTO-0.4NZFO composite film prepared by our 0–3 process indeed changes abruptly as expected, exhibiting a magnetoelectric coupling strength as large as 9.23% comparable to conventional 1–3 multiferroic composite films^5,14^. Compared with the magnitudes of the change in the magnetization of 0.8BTO-0.2NZFO and 0.4BTO-0.6NZFO composite thin films, it is evidenced that the magnetization of 0.6BTO-0.4NZFO composite is much higher. It indicates that only when the NZFO content is controlled in the region of x = 0.2–0.4, which locates between the percolation thresholds in two directions with the formation of 1–3 structure, can the magnetoelectric coupling strength of the multiferroic composites achieve a satisfactorily high level. More importantly, it has been verified that the 1–3 multiferroic composite films with strong magnetoelectric coupling can be easily prepared via a traditional 0–3 process by tailoring the anisotropy of the percolation threshold of composite films.

It should be noted that the grain sizes as well as the defects in composite films are changeable depending on the volume fraction of the components, according to our previous researches^23–25^. Nevertheless, the increase of NZFO grain size can be considered as a result of increasing ferrite volume. In particular, this work mainly focuses on the different percolative behaviors of the same BTO-NZFO composite thin films under electric and magnetic fields with different directions. In this scenario, the grain sizes and defects as well as their effect are supposed to be the same. Therefore, the influence of grain size and defects in lattices can be excluded in the course of interpreting the different percolative behaviors of the thin films under in-plane and out-of-plane external fields.

## Methods

**Figure 6:**
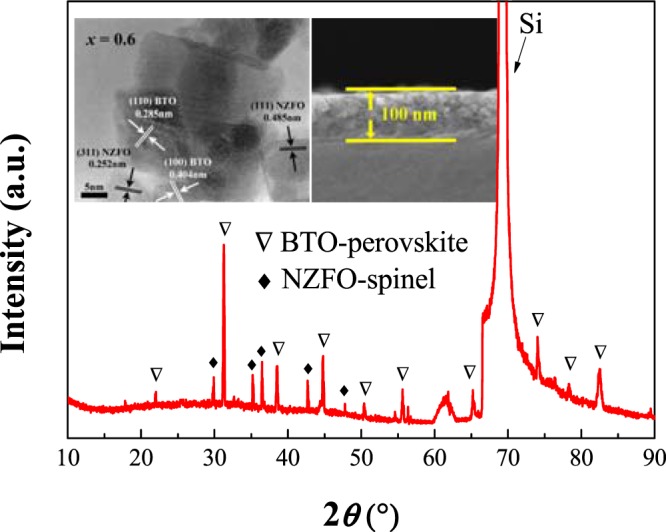
XRD pattern, TEM and cross-sectional SEM images of 0.4BTO-0.6NZFO composite thin film.

The BTO-NZFO composite films were prepared by magnetron sputtering as described in previous work. When the thicknesses of the BTO-NZFO composite films were controlled as 100 nm, the experimental compositions of the composite films were very closed to the nominal one (Table S1 in Supporting Information), and the grain sizes of BTO and NZFO are distributed in the range of 23.9–15.8 nm and 14.3–20.0 nm, respectively, as shown in Fig. 6^19–21^.

The electrical and magnetic properties of the composite films were tested with HP4292A-LRC Agilent 4292A Precision Impedance Analyzer and MPMS-XL-5 Magnetic Measurement System manufactured by Quantum Design Instruments, USA.

## Supplementary information

Supplementary Information
