# Supplementary material for: Anisotropy of Percolation Threshold of BaTiO3-Ni0.5Zn0.5Fe2O4 Composite Films
Source: Sci Rep. 2019 May 27;9:7855. doi: 10.1038/s41598-019-44328-7 (PMC6536557; doi:10.1038/s41598-019-44328-7)
Supplement: Supplementary file 1 — Supplementary Information [file 41598_2019_44328_MOESM1_ESM.docx]

Supplementary Information

**Anisotropy of Percolation Threshold of BaTiO_3_-Ni_0.5_Zn_0.5_Fe_2_O_4_ Composite Films**

Yu Tang^1,3^, Ruixin Wang^1^, Yi Zhang^2,^*, Shun Li^1^, Piyi Du^3^,*

1. Department of Materials Science and Engineering, College of Aerospace Science and Engineering, National University of Defense Technology, Changsha 410073, China

2. Department of Physics, College of Liberal Arts and Sciences, National University of Defense Technology, Changsha 410073, China

3. State Key Laboratory of Silicon Materials, School of Materials Science and Engineering, Zhejiang University, Hangzhou 310027, China

Email: zhangyi1983@zju.edu.cn, [dupy@zju.edu.cn](mailto:dupy@zju.edu.cn)

1.Table S1. The ratio (%) of the constituents of (1-x)BTO-xNZFO composite thin films. The nominal composition also are given in brackets.

| x | Ba | Ti | Ni | Zn | Fe |
| --- | --- | --- | --- | --- | --- |
| 0.1 | 40.97 (42.86) | 43.86 (42.86) | 2.29 (2.38) | 2.44 (2.38) | 10.44 (9.52) |
| 0.2 | 38.01 (36.36) | 35.79 (36.36) | 4.35 (4.55) | 4.32 (4.55) | 17.53 (18.18) |
| 0.3 | 31.68 (30.43) | 33.22 (30.43) | 7.11 (6.52) | 7.15 (6.52) | 20.84 (26.09) |
| 0.4 | 25.38 (25.00) | 24.32 (25.00) | 7.98 (8.33) | 8.64 (8.33) | 33.68 (33.33) |
| 0.5 | 19.01 (20.00) | 19.57 (20.00) | 9.51 (10.00) | 9.88 (10.00) | 42.03 (40.00) |
| 0.6 | 15.45 (15.38) | 15.52 (15.38) | 11.62 (11.54) | 12.03 (11.54) | 45.38 (46.15) |
| 0.7 | 10.68 (11.11) | 11.55 (11.11) | 12.57 (12.96) | 12.52 (12.96) | 52.67 (51.85) |
| 0.8 | 7.32 (7.14) | 7.25 (7.14) | 14.01 (14.29) | 14.7 (14.29) | 56.71 (57.14) |
| 0.9 | 3.46 (3.45) | 3.49 (3.45) | 15.52 (15.52) | 15.69 (15.52) | 61.85 (62.07) |
